# Editorial: Targeting tumor vasculature to enhance cancer immunotherapy

**DOI:** 10.3389/fonc.2023.1199811

**Published:** 2023-04-18

**Authors:** Jieying Yang, Li Fu, Toshiro Shirakawa, Tong Xiang

**Affiliations:** ^1^ State Key Laboratory of Oncology in South China, Collaborative Innovation Center for Cancer Medicine, Guangdong Key Laboratory of Nasopharyngeal Carcinoma Diagnosis and Therapy, Sun Yat-sen University Cancer Center, Guangzhou, China; ^2^ Guangdong Provincial Key Laboratory of Regional Immunity and Diseases, Department of Pharmacology and Shenzhen International Cancer Centre, Shenzhen University School of Medicine, Shenzhen, China; ^3^ Department of Urology, Kobe University Graduate School of Medicine, Kobe, Japan; ^4^ Department of Advanced Medical Science, Kobe University Graduate School of Science, Technology and Innovation, Kobe, Japan

**Keywords:** cancer immunotherapy, tumor vascularization, vascular normalization, endothelial cell anergy, immunosuppressive molecules

## Background

Cancer immunotherapy is an innovative treatment for tumors at present. In 2013, Science announced tumor immunotherapy as the technological breakthrough of the year (1). In almost 10 years of clinical trials, although immunotherapy represented by PD-1 monoclonal antibody drugs has shown obvious efficacy in patients with different types of cancer, their objective response rate (ORR) has only been about 20%, which eventually leads to disease progression (Chen et al.). Thus, new approaches that improve the clinical benefits of tumor immunotherapy are urgently needed.

The sufficient infiltration of immune effector cells and immunomodulator-related molecules used in tumor immunotherapy through tumor vascularization is a prerequisite for tumor immunotherapy response (2–4). However, the vasculature of tumors is highly abnormal and dysfunctional. Consequently, immune effector cells have an impaired ability to penetrate into solid tumors and often exhibit compromised functions. Tumor vascular normalization, overcoming tumor endothelial cell anergy, and the blockade of immunosuppressive molecules are current efforts in the targeting of the vasculature of tumors with the aim of improving the efficacy of cancer immunotherapy.

## Tumor vascular normalization

Given that the abnormal tumor vasculature is highly permeable, leaky, and tortuous with low perivascular coverage, which impairs blood flow and limits the immune cells and antibodies (5), strategies that normalize these aberrant blood vessels may therefore improve intertumoral immune cell infiltration and facilitate their antitumor activities. Multiple therapeutic strategies have been developed to normalize the tumor vasculature by tightening the endothelial cell junctions and improving pericyte coverage (6). Appropriate low-dose antiangiogenic therapy against VEGF/VEGFR was found to induce tumor vascular normalization, resulting in improved delivery of drugs and oxygen to targeted cancer cells (7). Accordingly, Fan et al. demonstrated that low-dose anlotinib can induce tumor vascular normalization and improves anti-PD-1 therapy. Zheng et al. further highlighted the recent advances of antiangiogenic immunotherapies in preclinical and clinical settings to solidify the concept that vascular normalization triggered by vasculature-targeting strategies potentiates cancer immunotherapy. Studies of murine and human tumors have identified the onset of normalization, typically 1–2 days after commencement of therapy, followed by an eventual “closure” of the normalization window (8). This opening window opportunity for designing controlled stepwise cancer cell death and immunological augmentation have been reviewed in detail by Swamy. However, these features of vascular normalization by low-dose antiangiogenic therapy strategies were eventually lost and replaced by pronounced vascular regression (9). Genetic approaches to normalization [such as promoting endothelial cell quiescence (e.g., PHD2 knockdown (10)) and enhancing vascular function (e.g., RGS5 knockdown (11))] give rise to a more prolonged normalization phenotype, in the absence of dramatic vessel regression. Li et al. established a hypoxia and angiogenesis prognostic model (HAPM) that has good predictive efficiency for PD-1 expression and T-cell exclusion, suggesting that this model may be utilized to forecast the genes for vascular normalization and the benefits of immunotherapy. In addition, Akter et al. discussed the therapeutic prospects of targeting heme and mitochondrial respiration in normalizing tumor vasculature, which provides a new theoretical basis for future research on the combination of vascular normalization and immunotherapy.

## Overcoming endothelial cell anergy

Abnormal tumor vasculature can also form a condition of inflammatory signals resulting in diminished leukocyte–vessel wall interactions and, therefore, decreased inflammatory infiltration, a process referred to as “endothelial anergy” (12). This interaction is mediated by cell adhesion molecules on both leukocytes and endothelium, such as intercellular adhesion molecule-1 (ICAM-1, CD54), vascular cell adhesion molecule-1 (VCAM-1, CD106), and E-selectin (CD62E) (13, 14). Rodriguez et al. provided insight into the mechanisms regulating peripheral node addressin (PNAd) biosynthesis in tumor endothelial cells and provided another platform to enhance its expression to support a continual influx of immune cells, sustaining antitumor immunity.

## Blockade of immunosuppressive molecules

Beside the endothelial cell anergy, abnormal tumor vasculature can express a range of inhibitory molecules, thereby creating a barrier for immune cells to infiltrate into the tumor tissue. Galectin 1 (15), the FAS ligand (FASL) (16), PD-L1 (17), and indoleamine 2, 3-dioxygenase(IDO) (18) were found to be selectively expressed in the vasculature of various malignancies, resulting in limited infiltration by activated T cells. Ileiwat et al. reviewed the mechanistic immunosuppressive role of the tumor vasculature and potential nanoparticle-mediated therapeutic strategies.

## Conclusion

In solid tumors, blood vessels are abnormal and dysfunctional, and thus immune effector cell infiltration is impaired. Although targeting the tumor vasculature has been shown to improve the efficacy of cancer immunotherapies, recent studies suggest that enhanced immune stimulation also, in turn, improves tumor vascular normalization (19, 20). A more comprehensive understanding of the crosstalk between the immune system and tumor vasculature can provide new strategies for treating human cancers.

## Author contributions

TX and JY drafted the manuscript. LF and TS revised the manuscript. All authors approved the submission.
